# Sub-national assessment of inequality trends in neonatal and child mortality in Brazil

**DOI:** 10.1186/1475-9276-9-21

**Published:** 2010-09-03

**Authors:** Angelica Sousa, Kenneth Hill, Mario R Dal Poz

**Affiliations:** 1 Initiative for Global Health, Harvard University, Cambridge, USA; 2 Harvard Center for Population and Development Studies, Harvard University, Cambridge, USA; 3 Department of Human Resources for Health, World Health Organization, Geneva, Switzerland

## Abstract

**Objective:**

Brazil's large socioeconomic inequalities together with the increase in neonatal mortality jeopardize the MDG-4 child mortality target by 2015. We measured inequality trends in neonatal and under five mortality across municipalities characterized by their socio-economic status in a period where major pro poor policies were implemented in Brazil to infer whether policies and interventions in newborn and child health have been successful in reaching the poor as well as the better off.

**Methods:**

Using data from the 5,507 municipalities in 1991 and 2000, we developed accurate estimates of neonatal mortality at municipality level and used these data to investigate inequality trends in neonatal and under five mortality across municipalities characterized by socio-economic status.

**Results:**

Child health policies and interventions have been more effective in reaching the better off than the worst off. Reduction of under five mortality at national level has been achieved by reducing the level of under five mortality among the better off. Poor municipalities suffer from worse newborn and child health than richer municipalities and the poor/rich gaps have increased.

**Conclusion:**

Our analysis highlights the importance of monitoring progress on MDGs at sub-national level and measuring inequality gaps to accurately target health and inter-sectoral policies. Further efforts are required to improve the measurement and monitoring of trends in neonatal and under five mortality at sub-national level, particularly in developing countries and countries with large socioeconomic inequalities.

## Introduction

Reducing child mortality by two thirds between 1990 and 2015 is the target of the fourth Millennium Development Goal (MDG). Recent analyses indicate that there has been major progress towards this goal. Current estimates show that the global under five mortality rate (U5MR) dropped from 93 per 1000 live births in 1990 to 72 per 1,000 in 2006 [1].

The targets to measure the progress towards the achievement of the Millennium Development Goals (MDGs) assess the progress of countries at the national level. However, national averages could be misleading, particularly in countries with great inequalities, as progress could be attained by improving the health of the well off while neglecting the health off the worst off. A recent report to monitor progress towards the attainment of the MDGs in 68 priority countries, which account for 97% of all maternal and child deaths, found that the poorest 20% of the population are less likely to be covered by effective interventions capable of preventing most maternal and child deaths than their wealthier counterparts [2]. It is therefore crucial to produce within country estimates to properly monitor progress of the MDGs and share countries' experience on policies and interventions that have been successful in reducing health inequalities and improving the health of the poor.

In the Latin America and Caribbean Region, the large inequalities in living conditions within countries together with the increase in the percentage of U5MR attributed to neonatal mortality jeopardize the MDG-4 target [3]. In Latin America, neonatal mortality represents more than half of overall infant mortality and 42% of under five deaths, and most of the neonatal deaths happen during the early neonatal period [4].

Brazil is the largest economy in Latin America [5] and it is considered to be one of the five most important emerging economies of the world along with Mexico, China, India and South Africa [6]. However, despite Brazil's economic achievements, it is still at the top of the list of countries with the highest income inequalities [7]. The poorest 20 percent earn 2 percent of the income while the richest 20 percent earn 63 percent (figures correspond to 2005, estimated from Pesquisa Nacional por Amostra de Domicilios, 2005) [5].

Brazil's national territory is divided administratively into 5 regions, 27 states and 5,507 municipalities (in 2000). There are wide variations in population, wealth, climate and size across geographical areas. For example, 25% of the municipalities have population below 5,000 inhabitants, while a few have populations above 1,000,000 inhabitants (the largest being the municipality of Sao Paulo with a population of 10.4 million).

In recent decades Brazil has achieved very important health gains [8] and is on track to achieve most of the MDG targets at the national level [9]. National level estimates show major progress in the reduction of U5MR. Between 1990 and 2005 child mortality dropped from 53.7 to 28.7, a declined of 46.6% and infant mortality dropped from 33.7 to 21.1, a declined of 37.4%, a drop of more than one third during the period, suggesting that Brazil is on track to reach the MDG-4 target by 2015 [9].

The health sector reform of the 1990's and the public health and inter-sectoral interventions implemented by the Brazilian government have contributed to the reduction of U5MR.

During the last two decades Brazil has undergone a series of health reforms aimed at: 1) providing universal access to health services free of charge to the entire population (Sistema Unico de Saude (SUS)- Unified Health System), and 2) decentralizing the decision making to the municipality governments (one level below the state). This resulted in the expansion of services, the most significant being the Family Health Program (described in Additional file 1: Appendix 1) (PSF), focused on providing and improving primary health care, particularly maternal and child care, among the poor [10-12]. Other public health and inter-sectoral programs have also been introduced to reduce infant and child deaths, such as access to clean water and sanitation, education of the mothers and immunization coverage. In addition, the federal government launched other programs to fight against hunger and poverty [13], such as the Zero Hunger program (Fome Zero) (described in Additional file 1: Appendix 2) [14] and the cash transfer program (Bolsa Familia) (described in Additional file 1: Appendix 3) [15]. In 2006, 99% of 1 year olds were covered by immunization of measles vaccine (MCV), diphtheria and tetanus toxoid and pertussis vaccine (DPT3), hepatitis B vaccine (HepB) and haemophilus influenzae type b (Hib3) and 92% of neonates were protected at birth against neonatal tetanus (PAB) [16].

However, despite the efforts made to improve access to health care services for the poor and the policies implemented to reduce child mortality, in practice there remain major health inequalities. The poorest region (Northeast) had the highest under five mortality rate of 39 per 1000 live births in 2005, whereas richer regions (South and Southeast) had the lowest child mortality rates of around 18.5 per 1000 live births [9]. Moreover, in 2004, 6% of the children less than five years old in the poorest region died from diarrhea, compared to 2% of the children in richer regions [17]. An evaluation of three maternal and child health programs found less access to health care among the poor [18]. A recent study also found that poorer municipalities had a lower proportion of deliveries attended in health facilities (76%) and a lower proportion of pregnant women covered by antenatal care (54%) than richer municipalities (for which the corresponding figures are 91% and 71% respectively) [19]. This is particularly important as most of the deaths of newborns are directly related to inadequate care during and after pregnancy and child birth [4,20].

Although the challenges involved in reducing child mortality are widely recognized, there is not enough evidence currently available to monitor whether newborn and child health interventions have been successful in reaching the poor at municipality level (the post-reforms level of decision making). Most of the existing evidence in neonatal and child mortality shows differences across regions or states [9,21-24] but very few analyses have been undertaken to monitor inequality trends across municipalities throughout the country [25,26]. Furthermore, evidence suggests that particularly in the poorest regions (North and Northeast), national civil registration data (the only source of information to produce municipal level estimates) are not an accurate source of information on infant deaths [27-30].

In this paper, we develop accurate estimates of neonatal mortality at the municipality level. We then use these estimates to measure inequality trends in neonatal and under five mortality across municipalities characterized by their socio-economic status, in a period where major health reforms and several pro poor policies were implemented in Brazil focused on decreasing child and neonatal mortality. We then infer whether these policies and interventions in newborn and child health have been successful in reaching the poor as well as the better off in Brazil.

## Data and methods

To analyze inequality trends in under five and neonatal mortality at the municipality level we constructed a dataset that compiles information on mortality rates and socioeconomic indicators for the 5,507 Brazilian municipalities for 1991 and 2000.

The most recent year of analysis is 2000, as it is the year with the most complete and accurate data to monitor differentials in child mortality at municipality level. Brazil has two systems to monitor vital events: the Mortality Information System (SIM) and the Information System on Live Births (SINASC). Several evaluations suggested however that neither system is complete enough to monitor differentials in infant and child mortality [27-30]. It is estimated that the Mortality Information System (SIM) [31] underreports 25% of deaths in the Northern Region and 29% in the Northeastern Region [28]. Similarly, the coverage of the Information System on Live Births (SINASC) [32] is only 73% in these two regions compared to the national coverage of 93% [33]. These inaccuracies are likely to be higher among children under one year of age and in municipalities with less than 50,000 inhabitants [29]. For these reasons we used Census data in this analysis.

Data on the under five mortality rate (described in Additional file 1: Appendix 4) per 1000 live births, estimated from the 1991 and 2000 population Census of Brazil, were obtained from the Institute of Applied Economic Research (IPEA), which compiles and produces publicly available data of socio-economic indicators of the municipalities [34].

The neonatal mortality rates per 1000 live births are a predictive estimate of the under five mortality data. First, we produced sub-national rates of neonatal mortality and child mortality by applying direct life table methods to birth histories from the various Demographic and Health Surveys (DHS). DHS is a (generally nationally representative) survey designed to collect detailed information on social and demographic characteristics and maternal and child health over a sample of women aged 15 to 49 years old in developing countries. The DHSs of Brazil used in this study were conducted in 1986, 1991 and 1996 and are sub-nationally representative: 1) 1986 is representative at regional level; 2) 1991 was conducted in the North-eastern region and is representative at the state level; and 3) 1996 is representative at the regional level and in four states in the North-eastern region, Rio Grande do Norte, Bahia, Ceará and Pernambuco [35-37]. Second, we used the rates produced from DHS to investigate the relationship between neonatal and under five mortality per 1000 live births at sub-national level using a log-log regression model. In addition, we included binary variables in the models to control for the different years of DHS. Finally, we extrapolated these relationships to predict neonatal mortality rates for the municipalities of the Southern and Northern Regions using the estimates of under five mortality rates (produced from the population Census) at municipality level for 1991 and 2000.

Data on the proportion of population below the poverty line (described in Additional file 1: Appendix 5), were obtained from IPEA [14]. These data were then used to group municipalities by poverty quintiles, where the fifth quintile represents the poorest 20% of municipalities and the first quintile the richest 20%. We also categorize municipalities as poor (the poorest 40%, 2,202 municipalities) or non-poor (the remaining 60%, 3,305 municipalities). The first two quintiles (40% of the municipalities) were considered as poor as they comprise the municipalities with more than 50% of their population below the poverty line.

To monitor whether improvements reached the worst off as well as the better off populations, there are two measures of the extent of mortality inequality. The first one, the relative measure, defined as the ratio of child mortality in the poorest quintile to the richest quintile; and the second one, absolute measure, defined as the difference in mortality between the poorest and richest quintile [38]. We also constructed an index to classify municipalities in four categories depending on the under five mortality reduction between 1991 and 2000 and the level of under five mortality in 2000 to identify the municipalities that require urgent policy interventions.

## Results

The results are presented in two subsections (and were produced using STATA 9 and ArcGIS 9.3 [39,40]). First, we show how we produced the data of neonatal mortality at municipality level for 1991 and 2000. Then, using these data and the existing data on under five mortality, we present the results of inequality trends in neonatal and child mortality across poor and non-poor municipalities in Brazil.

### a. Prediction of neonatal mortality

The log-log relationships between neonatal and under five mortality across sub-national units in Brazil are presented in Table 1. We found two different relationships; one for the three southern regions and a second one for the North and Northeast Regions. For the northern regions, a one percent increase in U5MR is associated with a 0.79 percent increase in NNMR, while for the southern regions, a one percent increase in U5MR is associated with 0.94 percent increase in NNMR. For the Southern Regions the model explains 94% of the total variance while for the Northern Regions the model explains 61%. It is likely that there are other socioeconomic characteristics (such as education of the mother) that were not taken into account in the model for the Northern Regions that are likely to explain the remaining variance. None of the dummy variables included in the models to control for differences in the survey years were significant.

**Table 1:** Log-log regressions of neonatal mortality on under five mortality among sub-national areas DHS 1986, 1991, 1996

Variable name	Northern Regions	**Southern Regions **^b^
Ln of U5MR	0.786**	0.940**
	(4.091)	(5.113)
Years^a^		
Dummy year 1991	-0.076	---
	(-0.472)	---
Dummy year 1996	-0.064	-0.128
	(-0.368)	(-1.335)
_cons	-0.134	-0.373
	(-0.148)	(-0.496)
R2	0.615	0.942
sample	17	9

^a ^the reference year is 1986, ^b ^for the Southern regions there is no DHS data in 1991 as it was conducted among the states of the Northeast Region. Statistical significance with a * p < 0.05; ** p < 0.01; *** p < 0.001

We used these relationships to predict neonatal mortality from existing estimates of under five mortality at municipality level for 1991 and 2000. We compared the averages of our predicted estimates of neonatal mortality by region with the numbers produced for the same year by two official sources of information; the Brazilian monitoring report on the MDGs, 2004 [25] and the 2008 Report on Basic Health Indicators (Indicadores Basicos para Suade no Brasil, 2008) (see Table 2) [17]. We found that our predicted estimates are very similar to the numbers presented in these two reports, which are corrected from the underreporting of deaths in the Northern Regions and used different methods and sources of information to produce their estimates (methods described in the notes of Table 2). This consistency implies that our predicted estimates of neonatal mortality are reasonable and can be used in the analysis presented in the following section. The advantage of our estimates of neonatal mortality is that they can be disaggregated at the municipal level which is the level where the decisions on the administration and provision of services are made.

**Table 2:** Comparisons of estimates of neonatal mortality with the estimates of two official sources of information 1) the Brazilian monitoring report on the MDGs 2004 and 2) the 2008 Report on Basic Health Indicators

Region	Indicadores Basicos para saude no Brasil Report (2000)*	MDGs Report NNM (2001)**	Predicted NNM (2000)	
			Mean	[95% CI]
NE	24.5	26.3	25.5	[25.2, 25.7]
N	18.7	18.4	18.3	[17.8, 18.9]
CW	14.2	14.6	14.2	[13.9, 14.6]
SE	12.3	12.5	13.4	[13.1, 13.7]
S	10.9	10.6	10	[9.7, 10.1]

* Estimates are produced using census data for some states [27] and for the remaining states estimates are produced using data for the Mortality Information System (SIM) and from the Live births Information System (SINASC).

** Estimates are produced combining census data and survey data of the Pesquisa Nacional por Amostra de Domicílio - PNAD.

### b. Inequalities in neonatal and under five mortality at sub-national level

**Figure 1:**
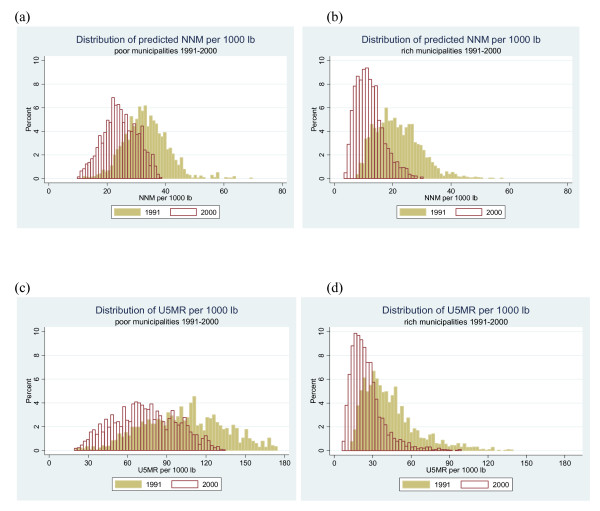
**Distribution of under five mortality and predicted neonatal mortality per 1000 lb by poor and non-poor municipalities in 1991 and 2000**.

The distributions of the predicted neonatal mortality and under five mortality across poor and non-poor municipalities are shown in Figure 1 a, b, c and 1d. In general, we found that between 1991 and 2000 there has been a decline in neonatal and under five mortality across poor and non-poor municipalities. Despite these declines, in 2000, poorer municipalities still have much higher rates of neonatal and under five mortality than richer municipalities.

**Figure 2:**
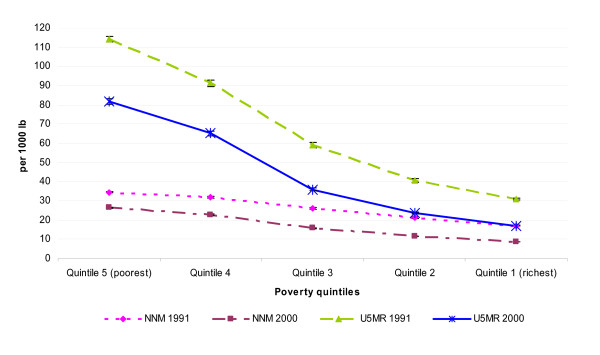
**Mean of under five mortality and predicted neonatal mortality per 1000 lb by poverty quintiles, Brazilian municipalities in 1991 and 2000**.

We looked in more detail at these differences by disaggregating the data into the five poverty quintiles. The average of the predicted neonatal mortality and under-5 mortality across municipalities grouped by poverty quintiles are shown in Figure 2 and in Table 3. Between 1991 and 2000, the averages of neonatal and under five mortality have decreased steadily across all socioeconomic groups. However, we found great inequalities across economic groups: the poorest municipalities had higher neonatal and under five mortality than any other economic quintile and this problem has not changed over time. For example, the neonatal mortality among the poorest municipalities in 2000 (26.4 per 1000 lb) is similar to the average national rate of this indicator in 1991 and to the national rates of low income countries like Eritrea (24 per 1000 lb) and Kenya (28 per 1000 lb), while the average rate among the richest municipalities (8.7 per 1000 lb) is similar to the national rates of upper-middle-income countries like Mexico and Romania [20].

**Table 3:** Inequality trends in under five mortality and predicted neonatal mortality per 1000 lb by poverty quintiles in 1991 and 2000

Poverty quintiles	NNM per 1000 lb*				U5MR PER 1000 lb*				Decrease rate 1991-2000	
	1991		2000		1991		2000			
	Mean	95% CI	Mean	95% CI	Mean	95% CI	Mean	95% CI	NNM	U5MR
Quintile 5 (poorest)	34.54	[34.16, 34.92]	26.40	[26.07, 26.73]	114.24	[112.64, 115.85]	81.87	[80.52, 83.21]	24%	28%
Quintile 4	31.89	[31.43, 32.35]	22.71	[22.39, 23.04]	91.38	[89.64, 93.13]	65.21	[63.89, 66.53]	29%	29%
Quintile 3	26.27	[25.82, 26.73]	15.79	[15.51, 16.07]	59.04	[57.59, 60.48]	35.98	[35.09, 36.87]	40%	39%
Quintile 2	21.35	[20.98, 21.72]	11.62	[11.43, 11.81]	40.65	[39.78, 41.52]	23.52	[23.08, 23.96]	46%	42%
Quintile 1 (richest)	17.07	[16.77, 17.37]	8.69	[8.53,8.84]	30.88	[30.27, 31.49]	17.06	[16.74, 17.38]	49%	45%
National	26.22	[25.98, 26.47]	17.04	[16.83, 17.25]	67.23	[66.21, 68.25]	44.72	[43.94, 45.51]	35%	33%

**Inequalities**										

**Q5/Q1 Ratio**	2.0		3.0		3.7		4.8			
**Q5-Q1 Differential**	17.5		17.7		83.4		64.8			

* National estimates differ from official sources due to differences in the sources of information.

From the relative ratio between the poorest 20 percent and the richest 20 percent of municipalities, we found that the inequity gaps have also increased in the period analyzed. The poorest municipalities had double the neonatal mortality of the richest municipalities in 1991 and this gap has increased to 3 times higher in 2000. These gaps are even larger in under five mortality: in 1991 the poorest municipalities had U5MRs 3.7 times greater than the richest municipalities and this gap increased to almost five times more in 2000. This implies that the policies and interventions introduced in the late 1980 s and 1990 s are failing to improve the relative position of the poor municipalities. The absolute differences show that the neonatal mortality between the poorest and richest municipalities differed by 17.5 deaths in 1991 and 17.7 in 2000, while the difference in under five mortality between these two groups fell from 83 deaths in 1991 to 65 in 2000.

In terms of the decline experienced between 1991 and 2000 at national level, there was a decline of 33% in under five mortality suggesting that Brazil is on track to meet the MDGs' child health target by 2015. However, this decline is not homogenous across poverty quintiles. We found an increasing gradient in the percentage decline by poverty quintiles, such that richer municipalities experienced a faster decline in under five and neonatal mortality than poorer municipalities. For example, the poorest municipalities experienced a reduction of 24% in neonatal mortality and of 28% in under five mortality, while the richest municipalities had a decline of 49% in neonatal mortality and of 45% in under five mortality.

**Figure 3:**
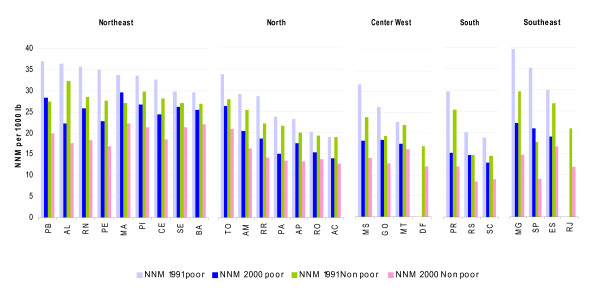
**Predicted neonatal mortality per 1000 lb between poor and rich municipalities within the States and Regions of Brazil in 1991 and 2000**.

Figure 3 shows that inequalities within regions and states are also very wide we found that inequalities in neonatal mortality between the poor and the rich have increased in a large majority of the states during the period analyzed. However, the highest inequalities were found in states belonging to richer regions -Southern Regions-. For example, we found that the states of Sao Paulo and Rio Grande do Sol had the highest inequalities in neonatal mortality in the country, with a poor non-poor gap of 2.4 and 1.7 in 2000 respectively. These are also the states with the highest inequalities across different socioeconomic indicators, which may explain our findings [41,42]. Sao Paulo for example, combines the poorest and the richest populations, thus it is not surprising that we also found the highest inequalities in neonatal mortality.

**Figure 4:**
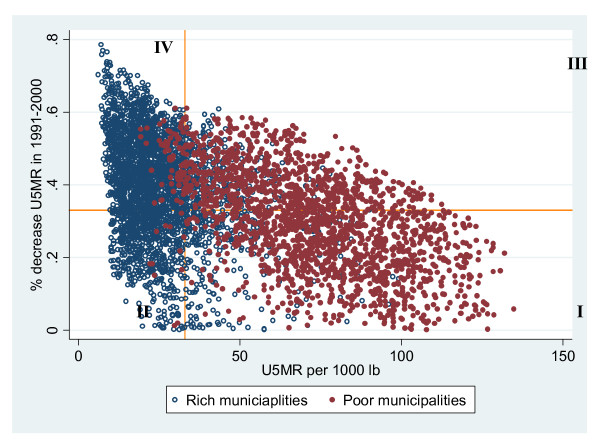
**Relationship between under five mortality reduction between 1991 and 2000 and the level of under five mortality per 1000 lb in 2000 by poor and non-poor municipalities, Brazil**.

We related the under five mortality reduction between 1991 and 2000 with the level of under five mortality in 2000 across poor and non poor municipalities (depicted in Figure 4). We added as cut-points the national values of both variables. The cut-point of 33% represents the national decline in under five mortality between 1991 and 2000 and the cut-point of 33 per 1000 lb represents the level of under five mortality at national level. From this plot we can observed four groups of municipalities: 1) group one, municipalities with high under five mortality -defined as rates above the national cut point of 33 per 1000 lb- and with low decline -defined as reduction below 33% national decline-; 2) second group, municipalities with low under five mortality -defined as rates below the 33 cut point- and low decline; 3) third group, municipalities with high under five mortality and high decline -defined as reductions above 33% national decline -; and 4) fourth group, municipalities with low under five mortality and high decline.

A large majority (81%) of municipalities in group one -low decline and high under five mortality- are poor municipalities, while almost all (96%) municipalities in group four -high reduction and low under five mortality- are rich municipalities.

**Figure 5:**
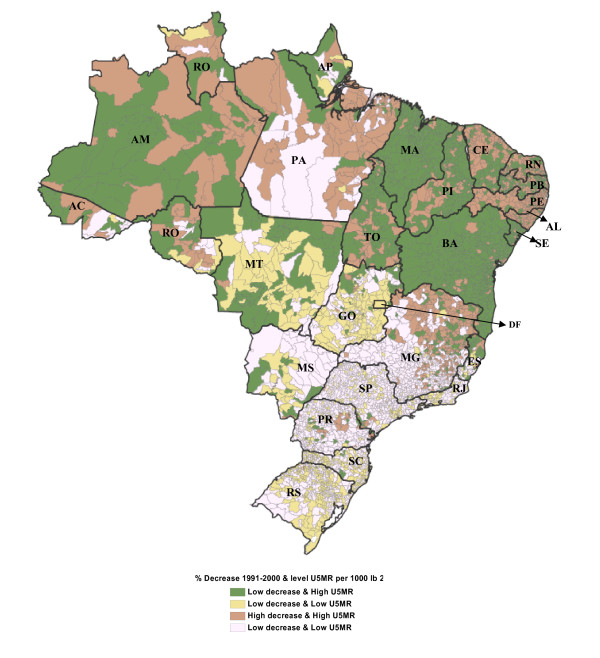
**Distribution of municipalities by categories of municipalities grouped by under five mortality reduction between 1991 and 2000 and the level of under five mortality in 2000**.

For policy purposes, we constructed an index (described in Additional file 1: Appendix 6) using the groups of municipalities of Figure 4 and mapped its distribution across municipalities to identify the critical geographical areas (see Figure 5). Not surprisingly, we found that the majority of municipalities in group one -low decline and high under five mortality- belong to the poorer regions (North and Northeast) where the majority of poor municipalities are concentrated. Specifically, a large majority (60%) of municipalities in this group are concentrated in four states of the Northeast Region: 24.4% in Bahia (identified as BA in Figure 3), 1,3% in Maranhão (MA), 10.4% in Piauí (PI), and 8.8% in Paraíba (PB), which are also the states were we found the highest levels of neonatal mortality among the poor in Figure 3.

However, we also found other states from wealthier regions with a significant number of municipalities in group one. For example, 47% of the municipalities in Mato Grosso (MS) and 33% of the municipalities in Espirito Santo (ES) were classified in group one despite the fact that these states belong to richer regions like the Center West and Southeast Regions.

Despite the socioeconomic conditions, there are some poor municipalities in the poorer regions that have been successful in reducing under five mortality to levels below 33 per 1000 lb (group three): these municipalities are mainly concentrated in the states of Pernambuco (PE), Rio Grande do Norte (RN), Alagoas (AL), and Ceará (CE) from the Northeast Region and Pará (PA) from the North Region.

## Conclusions

This study highlights the importance of conducting sub national level analysis to monitor progress on MDGs. Sub-national level analysis is also important in countries that have undergone decentralization reforms, specifically at the level where the decisions on the administration and provision of services are made in order to determine whether policies and interventions have been successful in improving the health of the population.

Several policy implications may arise from this study. As shown by the case of Brazil, the policies and interventions focused on improving newborn and child health have been more effective in reaching the better off than the worst off. However, the magnitudes of these inequalities are not shown by the national numbers. We found that the achievement in the reduction of under five mortality at national level has mainly been reached by reducing the level of under five mortality among the rich. Poor municipalities suffer from worse newborn and child health than richer municipalities and the poor/rich gaps have further increased, thus jeopardizing the possibility of reaching the MDG-4 target by 2015. This findings are consistent with the report to track progress on the attainment of the MDGs on child and maternal mortality (MDGs 4 and 5) by 2015 [2].

Most of the deaths between one year and five years old are related to infectious disease and could be averted by very cost effective interventions [43]. However despite the implementation of specific interventions to decrease child mortality in poor areas, still 6% of children under five in these areas die from diarrhea [17]. In addition, despite the implementation of actions to increase access to clean water and sanitation among the poor, still 83% of the households in rural areas do not have access to improved drinking water as compare to 9% of the households in urban areas [44]. It is therefore crucial to pursue intersectoral interventions to improve the socioeconomic conditions of poor municipalities.

In the other hand, most of the deaths of newborns could be prevented with access to adequate care during and after pregnancy and child birth [4,20]. However, a recent study found that poorer municipalities had lower proportion of deliveries attended in health facilities and proportions of pregnant women covered by antenatal care than richer municipalities, the differences being attributed to lack of human resources, qualified personnel, and local health facilities [19]. Thus, further efforts are required from municipal, state and federal authorities to make health systems more equitable and to identify the interventions that have the ability to reach the poor and reduce socioeconomic inequalities in maternal, newborn and child health.

These results contribute to a major understanding of inequalities in newborn and child health within Brazil. Brazil's MDG report 2007, has pointed out that policies and programs should be targeted to improve the health of the population in the poorest Regions (the North and Northeast) to address health inequalities [9]. In this study, we provide further evidence and found that not all the municipalities in these regions require additional policy interventions. In fact, despite being economically disadvantaged, some poor municipalities in the North and Northeast are performing very well and their policies and interventions can be used as an example for further actions by municipalities with similar socioeconomic conditions. Furthermore, the majority of the municipalities with low decline and high levels of under five mortality have also very high levels of neonatal mortality. These municipalities are concentrated in four states in the Northeast Region, but some are found in richer states and richer regions.

The conclusions drawn from this study should take into consideration the limitations of the data. The data used in this study are from 2000, which is the most recent year with valid estimates of child mortality at municipal level (as pointed out in the methods). It is therefore likely that the level of neonatal and child mortality in this study overestimate the current numbers. Although, several national and sub-national interventions have been implemented to reduce inequalities in neonatal and under five deaths across socioeconomic groups since 2000, the most recent information shows that in general, between 2000 and 2005, there have been relatively few improvements in neonatal and child health inequalities between the poorest and richest regions [9,17].

Data quality may have affected the pattern of our estimates of neonatal mortality in the Northern Regions. We have therefore performed a sensitivity analysis producing the estimates of neonatal mortality for the municipalities in the Northern Regions using the relationship of the Southern Regions (from Table 1). We found that the estimated neonatal mortality in the Northern municipalities is higher if the Southern relationship is used than the estimates when the Northern relationship is used. We also found that the patterns found across municipalities do not change and even more the inequalities are more accentuated when the Southern relationship is used. This implies that the results and conclusions found in this study do not change when using different extrapolation models.

We found that the model of the Northern Regions explains 61% of the total variance while for the Southern Regions it explains 94% of the variance. Although the lack of explanatory power of the model in the Northern relationship may have affected the quality of our estimates the sensitivity analysis demonstrates that the patterns found remain the same even when using different models.

Despite the limitations associated with the data, this study has highlighted some critical issues in terms of the persistent inequalities in neonatal and child mortality within Brazil. This is particularly important for most developing countries and countries with great inequalities, thus further efforts are required to improve the measurement and monitoring of trends in neonatal and under five mortality at sub-national level.

## Competing interests

The authors declare that they have no competing interests.

## Contribution of authors

AS conceived the study, conducted the analysis and interpretation of the data and drafted the paper. KH advised on the methodology, contributed to the interpretation of results and writing of the paper. MDP contributed to the interpretation of results. All authors read and approved the final manuscript.

## Supplementary Material

Additional file 1 **Appendices 1-6**. Click here for file

## References

[B1] United Nations Children's Fund (UNICEF) The state of the world's children 2008: Child survivle 2008 New York, USA, United Nations Children's Fund (UNICEF)

[B2] Boerma JT Bryce J Kinfu Y Axelson H Victora CG Mind the gap: equity and trends in coverage of maternal, newborn, and child health services in 54 Countdown countries Lancet 2008 371 1259 1267 10.1016/S0140-6736(08)60560-7 18406860

[B3] Pan American Health Organization Social protection in health schemes for mother, newborn and child populations: lessons learned from the Latin American Region 2008 Washington DC, Pan American Health Organization/World Health Organization (PAHO/WHO)

[B4] World Health Organization Neonatal and perinatal mortality: country, regional and global estimates 2006 Geneva, Switzerland, World Health Organization

[B5] World Bank World Development Indicators database 2008 2008 World Bank http://go.worldbank.org/X0BDGIJK30

[B6] G8 Summit Chair's Summary 2007 Heiligendamm, G8 Summit

[B7] World Bank Inequality and Economic Development in Brazil 2004 Washington DC: World Bank

[B8] Rehem de Souza R El sistema publico de salud Brasileno 2002 Minsterio da Saude Brasil

[B9] Objetivos de Desenvolvimento do Milênio Relatório nacional de acompanhamentocoordenação: Instituto de PesquisaEconômica Aplicada e Secretaria de Planejamento e InvestimentosEstratégicos and supervisão: Grupo Técnico para o acompanhamentodos ODM 2007 Brasilia, IPEA

[B10] Facchini LA Piccini RX Tomasi E Thume E Teixeira VA da Silveira DS Evaluation of the effectiveness of primary health care in South and Northeast Brazil: methodological contributions Cadernos de Saude Publica 2008 24 S159 S172 18660900 10.1590/s0102-311x2008001300020

[B11] Aquino R de Oliveira NF Barreto ML Impact of the Family Health Program on Infant Mortality in Brazilian Municipalities American Journal of Public Health 2009 99 87 93 10.2105/AJPH.2007.127480 19008516 PMC2636620

[B12] Departamento de Atenção Básica (DAB) Atenção Básica e a Saúde da Família (PSF) 2007 Ministerio da Saude Brasil http://200.214.130.35/dab/conheca_dab.php

[B13] Belik W Mauro G Brazil's zero hunger program in the context of social policy 2003 Unicamp

[B14] UNDP Brasil Pobreza e Fome: objetivo 1 - erradicar a extrema pobreza e a fome [Poverty and Hunger: objective 1 - to eradicate poverty and hunger] 2004 Brasil, UNDP Brasil, Universidade Federal do Rio Grande do Sul, Instituto de Desenvolvimento Humano Sustentável

[B15] Veras SF Soares S Medeiros M Guerreiro OF Cash transfer programmes in Brazil: impact on inequality and poverty. Working Paper No.12 2006 Brasilia, Brazil, International Poverty Center, UNDP/IPEA

[B16] World Health Organization World Health Statistics 2008 Geneva, Switzerland, World Health Organization

[B17] REDE Interagencial de Informação para a Saúde Indicadores básicos para a saúde no Brasil: conceitos e aplicações 2008 Brasil, Organizaçãon Pan-Americana da Saúde

[B18] Barros AJD Victora CG Cesar AJ Neumann NA Bertoldi AD Gwatkin DR, Wagstaff A, Yazbeck A Brazil: are health and nutrition programs reaching the neediest? Reaching the poor with health, nutrition, and population services what works, what doesn't, and why 2005 Washington, DC: The World Bank

[B19] Sousa A Canning D Dal Poz MR Evans BD Health service provision and social determinants: Evidence from maternal health in Brazil Forthcoming 2010

[B20] World Health Organization The World Health Report - Make Every Mother and Child Count 2005 Geneva, Switzerland, World Health Organization

[B21] Victora CG Barros FC Infant mortality due to perinatal causes in Brazil: trends, regional patterns and possible interventions Sao Paulo Med J 2001 119 33 42 10.1590/S1516-31802001000100009 11175624 PMC11159561

[B22] Matijasevich A Victora CG Barros AJD Santos IS Marco PL Albernaz EP Widening ethnic disparities in infant mortality in southern Brazil: Comparison of 3 birth cohorts American Journal of Public Health 2008 98 692 698 10.2105/AJPH.2006.093492 17761568 PMC2376998

[B23] Volpe FM Abrantes MM Capanema FD Chaves JG The impact of changing health indicators on infant mortality rates in Brazil, 2000 and 2005 Revista Panamericana de Salud Publica-Pan American Journal of Public Health 2009 26 478 484 20107701 10.1590/s1020-49892009001200002

[B24] Victora CG Matijasevich A Silveira MF Santos IS Barros AJD Barros FC Socio-economic and ethnic group inequities in antenatal care quality in the public and private sector in Brazil Health Policy Plan 2010 25 253 261 10.1093/heapol/czp065 20123940 PMC2889278

[B25] Technical Group for Monitoring the Millennium Development Goals Brazilian Monitoring Report on the Millennium Development Goals Institute of Applied Economic Research (IPEA) and National Institute of Geography and Statistic (IBGE) 2004 Brasilia, Brazil, Institute of Applied Economic Research (IPEA)

[B26] Duarte CM [Health policy effects on infant mortality trends in Brazil: a literature review from the last decade] Cad Saude Publica 2007 23 1511 1528 17572800 10.1590/s0102-311x2007000700002

[B27] Castro OJ Albuquerque FR Braga LI Projecao da populacao do Brasil por sexo e idade para o periodo 1980-2050. Estimativas anuais e mensais da populacao do Brasil e das unidades da federacao 1980-2020 Estimativas das populacoes municipais: Metodologia 2004 Rio de Janeiro, Instituto Brasileiro de Geografia e Estadistica (IBGE). Directoria de Pesquisas (DPE). Coordenacao de Populacao e Indicadores Sociais (COPIS)

[B28] Cardoso A International Microdata Scoping Studies Project: Brazil 2007 Rio de Janeiro, Economic and Social Research Council (ESRC)

[B29] de Andrade CL Szwarcwald CL [Socio-spatial inequalities in the adequacy of Ministry of Health data on births and deaths at the municipal level in Brazil, 2000-2002] Cad Saude Publica 2007 23 1207 1216 17486242 10.1590/s0102-311x2007000500022

[B30] Szwarcwald CL Leal MC de Andrade CL Souza PR Jr [Infant mortality estimation in Brazil: what do Ministry of Health data on deaths and live births say?] Cad Saude Publica 2002 18 1725 1736 12488900 10.1590/s0102-311x2002000600027

[B31] Ministério da Saúde Mortality Information System [Sistema de Informações sobre Mortalidade (SIM) [on-line]] 2008 Brasília, Ministério da Saúde

[B32] National Agency for Health Surveillance Sistema de Informações de Nascidos Vivos (SINASC) 2000 Ministry of Health Brazil

[B33] Cardoso AM Santos RV Coimbra CE Jr [Infant mortality according to race/color in Brazil: what do the national databases say?] Cad Saude Publica 2005 21 1602 1608 16158168 10.1590/S0102-311X2005000500035

[B34] Instituto de Pesquisa Economica Aplicada (IPEA) Dados macroeconomicos e regionais 2008 http://www.ipeadata.gov.br/

[B35] Sociedade Civil Bem-Estar Familiar no Brasil (BEMFAM) and Demographic and Health Surveys (DHS) Pesquisa nacional sobre demografía e saúde 1991 [Brazil. National demographic and health survey 1991] 1991 Rio de Janeiro/Calverton, MD, BEMFAM/Macro International Inc

[B36] Sociedade Civil Bem-Estar Familiar no Brasil (BEMFAM) and Demographic and Health Surveys (DHS) Pesquisa nacional sobre demografía e saúde 1996 [Brazil. National demographic and health survey 1996] 1997 Rio de Janeiro/Calverton, MD, BEMFAM/Macro International Inc

[B37] Sociedade Civil Bem-Estar Familiar no Brasil (BEMFAM) and Demographic and Health Surveys (DHS) Pesquisa nacional sobre demografía e saúde 1986 [Brazil. National demographic and health survey 1986] 1987 Rio de Janeiro/Calverton, MD, BEMFAM/Macro International Inc

[B38] Anand S Diderichsen F Evans T Vladimir M Wirth M Evans T, Whithead M, Diderichsen F, Bhuiya A, Wirth M Measuring Disparities in Health: Methods and Indicators Challenging inequities in health: from ethics to action 2001 Oxford University Press

[B39] Environmental Systems Research Institute (ESRI) ArcGIS (Geographic Information System software). [9.2] 2005 NY, USA, Redlands, CA: Environmental Systems Research Institute

[B40] StataCorp Stata Statistical Software. [9] 2005 College Station, TX: StataCorp LP

[B41] Szwarcwald CL de Andrade CLT Bastos FI Income inequality, residential poverty clustering and infant mortality: a study in Rio de Janeiro, Brazil Social Science & Medicine 2002 55 2083 2092 10.1016/s0277-9536(01)00353-7 12409122

[B42] Rocha S Poverty and Inequality in Brazil: The Depletion of the Distributive Effects of the Real Plan. [Working Paper No. 721] 2000 IPEA

[B43] Claeson M Gillespie D Mshinda H Troedsson H Victoria CG Knowledge into action for child survival Lancet 2003 362 323 327 10.1016/S0140-6736(03)13977-3 12892965

[B44] United Nations Children's Fund (UNICEF) Country, regional and global estimates on water & sanitation 2009 UNICEF

