# Supplementary material for: Sub-national assessment of inequality trends in neonatal and child mortality in Brazil
Source: Int J Equity Health. 2010 Sep 3;9:21. doi: 10.1186/1475-9276-9-21 (PMC2944212; doi:10.1186/1475-9276-9-21)
Supplement: Additional file 1 — Appendices 1-6. [file 1475-9276-9-21-S1.DOC]

**Appendix:**

^1^ This program was implemented in 1994 to expand primary health care particularly to the municipalities that did not have primary health care facilities and to refer patients to more complex levels of care [1].

^2^ The aim of this program is to guarantee the access to basic food by making direct transfers to the poorest families to raise their consumption of food, distributing vitamins and iron supplements, among others [2]

^3^ This program provides financial aid to poor and indigent families conditional on attendance at school for children, updated immunization cards for children under six years old and regular visits to health center for breast feeding or pregnant women [3].

4 The under five mortality rates were estimated by applying indirect demographic methods to information from reports of women concerning the survival of their children ever born from the 1991 and 2000 population Census of Brazil [4], using the Coale and Demeny West family table to convert probabilities of dying to mortality levels [5].

^5^ Defined as the proportion of population with a monthly family income per capita of less than 75$R, which is equivalent to half a minimum wage per capita in August 2000. The universe of population is the total number of people with a permanent address. This indicator is constructed from the Population Census.

^6^ 1) group one, municipalities with high under five mortality -defined as rates above the national cut point of 33 per 1000 lb- and with low decline -defined as reduction below 33% national decline-; 2) second group, municipalities with low under five mortality -defined as rates below the 33 cut point- and low decline; 3) third group,

municipalities with high under five mortality and high decline -defined as reductions above 33% national decline -; and 4) fourth group, municipalities with low under five mortality and high decline.
